# Monitoring Asymptomatic Apical Periodontitis in Root‐Filled Teeth—Results From a One‐Year Follow‐Up Prospective Study

**DOI:** 10.1111/iej.70059

**Published:** 2025-11-13

**Authors:** M. Sayady‐Ölander, J. Östlin, V. S. H. Yu, C. Ulin, EndoReco, L. Bjørndal, V. S. Dawson, H. Fransson, F. Frisk, M. Markvart, F. Mota de Almeida, M. Pigg, D. Sebring, E. Wigsten, T. Kvist

**Affiliations:** ^1^ Public Dental Service Västra Götalandsregionen Sweden; ^2^ Faculty of Dentistry National University of Singapore Singapore City Singapore; ^3^ Endodontic Research Collaboration in Scandinavia Gothenburg Sweden; ^4^ Department of Endodontology Institute of Odontology, the Sahlgrenska Academy, University of Gothenburg Göteborg Sweden

**Keywords:** apical periodontitis, endodontics, monitoring, prospective cohort study, root‐filled tooth

## Abstract

**Aim:**

To prospectively investigate the incidence of symptoms and changes in the size of the apical bone destruction in symptom‐free root‐filled teeth with persistent apical periodontitis (AP) over 1 year without treatment.

**Methodology:**

Patients referred to two specialist endodontic clinics in Västra Götaland, Sweden, with asymptomatic periapical bone destructions (summing to ≤ 10 mm in diameter, when measured in two dimensions) on intraoral radiographs were invited. After informed consent, participants underwent cone‐beam computed tomography (CBCT). Patients with bone destructions beyond cortical boundaries were excluded. A 1‐year follow‐up included recording subjective symptoms, clinical findings and estimating bone destruction size.

**Results:**

Of 187 eligible patients, 171 (91.4%) participated. After CBCT, four were excluded, and 10 were lost to follow‐up. Among 157 patients, three developed symptomatic AP and were treated, and one tooth was lost due to fracture. Of the 153 patients with complete follow‐up, eight (5.2%) reported mild pain or discomfort but did not seek care. Lesion size changes were small (*M* = 0.059 mm), with the mean increasing from 7.25 mm (SD 2.11) to 7.31 mm (SD 2.33), which was not statistically significant.

**Conclusion:**

The findings align with previous studies, suggesting that patients with symptom‐free root‐filled teeth with apical periodontitis rarely experience exacerbation or significant increase in size of bone destruction in the short term. Longer follow‐up is needed to identify potential risk factors.

## 1 Introduction

In radiographic surveys of various populations, the frequency of apical bone destructions as a sign of persistent root canal system infection and inflammation in the periradicular tissues, apical periodontitis (AP) is a common finding (Pak et al. 2012; Jakovljevic et al. 2020; Tibúrcio‐Machado et al. 2021). The number of root‐filled teeth with AP has been estimated to be somewhere between 2.5 million and 3.6 million only in Sweden (Kvist 2001; Figdor 2002) and in the USA 117 million (Figdor 2002).

Because of the scarcity of well‐designed studies, the natural course of root‐filled teeth with signs of AP is largely unknown. In most cases, the infection and inflammation are likely to remain asymptomatic. But it happens that the inflamed area increases in size, root resorption occurs, or the patient suddenly develops symptoms in the form of swelling and toothache (Swedish Council on Health Technology Assessment 2010).

There is great variation and disagreement about how asymptomatic root‐filled teeth with AP should be managed in everyday clinical dentistry (Kvist 2001; Kvist and Hofmann 2023). From an academic point of view, it has been an axiom that these teeth should be treated (Kvist 2001). However, in general practice many root‐filled teeth with AP are left without treatment or systematic follow‐up (Petersson et al. 1991; Kvist et al. 2004; Olsson et al. 2024).

Studies have pointed out that it is in cases with smaller and medium‐sized lesions (approximately > 2 < 10 mm in diameter) that “no treatment” or “monitoring” are frequently selected as options for root‐filled teeth with AP among general dental practitioners (Reit and Gröndahl 1987; Kvist 2001; Kvist et al. 1994; Rawski et al. 2003; Olsson et al. 2024).

In specialist endodontic clinics, management of root‐filled teeth occupies about 50% of the clinical time (Abbot 1994; Kim 2014; Sebring et al. 2017). Retreatment is associated with costs in the order of €300–€2000, depending on the selected method (surgical or non‐surgical) and the need for new direct or indirect restoration (Wigsten et al. 2018). Patients who have subjective complaints from their root‐filled tooth need to actively seek treatment. However, for those who are asymptomatic, the necessity of treatment is questioned (Swedish Council on Health Technology Assessment 2010; Bergenholtz 2016; Kvist and Hofmann 2023).

The incidence of severe pain from asymptomatic root‐filled teeth with AP has been estimated to be low (1%–6%) in long‐term retrospective studies (Van Nieuwenhuysen et al. 1994; Yu, Messer, Yee, and Shen 2012). However, an increase in the severity of the radiographic appearance of the bone destruction (assessed as an increase in PAI score (Ørstavik et al. 1986)) was found to be approximately 52% over 4 years in a prospective study (Tsesis et al. 2013) while an increase in the size of the destruction was found in approximately 31% in a retrospective study over 4–21 years (Yu, Messer, Shen, et al. 2012).

Although the decision‐making problem is common and equally important for patients, dentists as well as third party payers, well‐designed prospective studies on the natural course of AP in root‐filled teeth are rare and evidence is needed.

## 2 Aim

The aim of this study was to systematically and prospectively investigate the clinical and radiographical course of symptom‐free root‐filled teeth with signs of AP over a 1‐year period.

## 3 Material & Methods

The study was designed as a prospective cohort study in which a group of patients with AP in root‐filled teeth is followed without intervention. Although the study was planned before the publication of specific guidelines for observational studies in endodontics, PROBE (Nagendrababu et al. 2023), the study design is substantially compatible with these.

### 3.1 Recruitment of Patients—Referral Assessment

As this was an exploratory observational cohort study without a predefined primary outcome or comparison groups, no formal sample size calculation was performed. The aim was to include as many eligible cases as possible to allow meaningful analysis.

Patients for the project were recruited from patients referred to the specialist clinics for endodontics, Public Dental Service, Västra Götalandsregionen, Gothenburg, and Mölndal, Sweden. The clinic's referral recipient, a specialist in endodontics, consecutively assessed incoming referrals for suitability in the study according to the criteria below. In the case of patients referred for more than one root‐filled tooth only one tooth per patient (the first tooth mentioned in the referral) was considered for the study.

### 3.2 Inclusion Criteria

- The patient should be substantially healthy with ASA Physical Status Class I and II (ASA n.d.).
- The tooth should have a root filling placed ≥ 4 years.
- The tooth should exhibit a clearly visible apical bone destruction, where the sum of two perpendicular diameter measurements ≤ 10 mm, on intraoral radiographs at a chairside evaluation.
- The tooth should be and should have been symptom‐free for at least 6 months before examination.

### 3.3 Exclusion Criteria

- The tooth has marginal attachment loss more than 6 mm with concomitant bleeding on probing.
- The patient has received a prescription of antibiotics for treatment of the tooth during preceding 6 months.
- The tooth is planned to receive a new filling or crown for any reason.

### 3.4 Recruitment of Patients—Clinical and Radiographic Examination

Patients judged eligible at referral assessment were examined by a specialist dentist or a postgraduate student in endodontics. In addition to a check of the referral information and the patient's medical history, there was a reassurance that the tooth was symptom‐free. Besides registration of tooth number, the clinical examination included percussion pain or tenderness, crown palpation pain or tenderness, apical palpation pain or tenderness, as well as probing of the gingival pocket for measuring depth (mm) and bleeding (yes or no) of the tooth in question. Furthermore, we notified the type of restoration, as a crown, an abutment in a bridge or a filling and in the case of the latter the number of filled surfaces (1–5).

Also, the examining dentist exposed two intra‐oral images, using the paralleling technique and with a difference in horizontal angulation of approximately 10° and PSP plates Vistascan plus (Durr Dental, Beitigheim‐Bissingen, Germany) for patients included until 2018 and the image sensor Planmeca ProSensor HD (Planmeca OY, Helsinki, Finland) thereafter.

The need for a new restoration was evaluated considering clinical and radiological findings indicating caries lesions or poor coronal restoration. At chairside, bone loss extent was assessed using available intra‐oral radiographs and the built‐in measurement tool in the Planmeca Romexis 5.1 software (Planmeca OY, Helsinki, Finland) to include only teeth with bone destructions where the sum of two perpendicular diameter measurements ≤ 10 mm in diameter.

If the patient met the inclusion criteria while none of the exclusion criteria were at hand, the patient was invited to participate in the study by giving verbal and written information. In case of willing to participate, patient needed to give a written consent and as a voluntary opportunity we offered a Digital Volume Tomography (CBCT) at the Specialist Clinic for Oral and Maxillofacial Radiology at Public Dental Service Västra Götalandsregionen. It was informed to the patient that if CBCT examination would show that the apical bone destruction broke through cortical constraints against anatomical structures such as the mandibular canal, maxillary sinus or nasal cavity, or if the destruction affects neighbouring teeth's periodontal or root system (resorptions), he or she would be excluded from participation in the study and suggested an appropriate intervention.

The majority of the CBCT images were exposed using the 3D Accuitomo 170 CBCT unit (J Morita, Kyoto, Japan) with a field of view (FOV) 4 × 4 cm, 360°, continuous exposure, exposure time 17.5 s, exposure parameters 80–90 kV and 4–7 mA. Voxel size 80 μm. Some CBCT images were obtained with Promax 3Dmid (Planmeca OY, Helsinki, Finland) with FOV 4 × 5 cm, pulsed exposure, exposure time 12 s, exposure parameters 90 kV and 5–10 mA. Voxel size 200 μm. In the CBCT examinations all images were analysed in the Multi Planar Reconstruction mode of Sectra PACS, for findings (Sectra PACS n.d.). A licensed oral and maxillofacial radiologist examined the images and gave a written radiological report about the presence, size and anatomical features of the pathological bone destruction. The report was received by the dentist who had executed the clinical and intraoral radiological examination.

### 3.5 Recalls

Included patients were scheduled for a 1‐year follow‐up clinical examination including two intraoral radiographs. The examinations were performed exclusively by one of the authors (MSO) on one occasion without repetition. At this visit, the patient was asked to report any events regarding the included tooth during the past year and to explicitly answer yes or no to whether any symptoms had occurred. The clinical examination was performed in the same way as on the baseline examination: percussion, pain or tenderness, crown and apical palpation pain or tenderness as well as bleeding on probing of gingival pockets was recorded as yes or no. Gingival pocket depth was given in millimetres.

### 3.6 Outcome Measures

The primary outcome was any recorded emergence of subjective and/or clinical symptoms and/or associated treatments.

The secondary outcome was other clinical events (caries, fractures, loss of filling) and associated interventions.

The third outcome was a change in the size of the apical bone destruction.

### 3.7 Evaluation of the Intraoral Radiographs

Two of the authors (MSO and JO) analysed all the intraoral radiographs (2 from baseline examination and 2 from follow‐up) on an Eizo Rx240 screen (Eizo Corporation, Ishikawa, Japan) with a resolution of 1600 × 1200 in a room with subdued lighting. The screens had built‐in sensors using the program RadiCS for calibration. The observers could adjust the brightness and contrast of the screen and recorded the following variables:

- The size of the bone destruction by measuring the extent of the bone destruction in two perpendicular directions; mesial to distal and superior to inferior and by including the estimated area over the tip of the root (Kvist and Reit 1999). As default, the observers measured on the orthoradial image, but if the periapical lesion was difficult to observe on this image, the eccentric image was chosen instead. In multi‐rooted teeth, measurements were made at the root with the largest bone destruction. The measurements were made with the built‐in measurement tool in Planmeca Romexis 5.1.
- Any presence of root resorption.
- Unfilled root canals.
- Any presence of fractured instrument in root canal.
- Any excess root filling material beyond the root apex (Overfilling).
- Any visible lumen apical to the root filling (Underfilling)
- The quality of root filling by assessing presence of voids or poor adaptation to root canal walls (judged as adequate or defective).

### 3.8 Observer Calibration and Evaluation of Observer Variation

Firstly, a session for calibration between the two observers took place. The intra‐oral images from 10 patients were analyzed jointly for consensus and agreement.

Secondly, among baseline intra‐oral radiographs 25 teeth were randomly selected. The two observers independently measured and recorded the above variables on two occasions separated by a minimum of 14 days.

### 3.9 Recording of Intra‐Oral Radiographs Variables

All baseline radiographs were randomly assigned in equal numbers to the two observers and assessed according to the previously described method. Approximately 2 months later, the 1‐year follow‐up radiographs were also randomly assigned and assessed using the identical procedure.

### 3.10 Ethical Considerations

The project has been evaluated and approved by the Ethics Review Board in Gothenburg, Sweden (2015‐06‐23). Diary number: 280‐15.

### 3.11 Statistical Methods

Continuous variables are described with mean, standard deviation (SD), median (Q1; Q3) and categorical variables with numbers and percentages.

Intra‐individual agreement in continuous variables (measurement of lesion size) was analysed with the distribution of the difference between the two observations, intra‐individual standard deviation (IISD), intraclass correlation coefficient (ICC). For analysis of systematic differences between the two measurements Wilcoxon's Signed Rank test was used.

Agreement analyses regarding dichotomous variables were described with percent agreement, kappa and Gwet's AC1, all with 95% confidence intervals (CI) and analyzed with the Sign test.

For analysis of change in continuous variables over time Fisher's non‐parametric permutation test for paired observations was used. Mean difference with 95% CI, *p*‐value and effect size are the main results. Effect size was estimated using the Cohen method (difference in mean/SD at the first timepoint). For comparison over time, the Sign test was used for categorical variables. All statistical tests were two‐sided and conducted at the 5% significance level.

For all analyses SAS Version 9.4, SAS Institute, Cary, NC, USA was used.

## 4 Results

**FIGURE 1:**
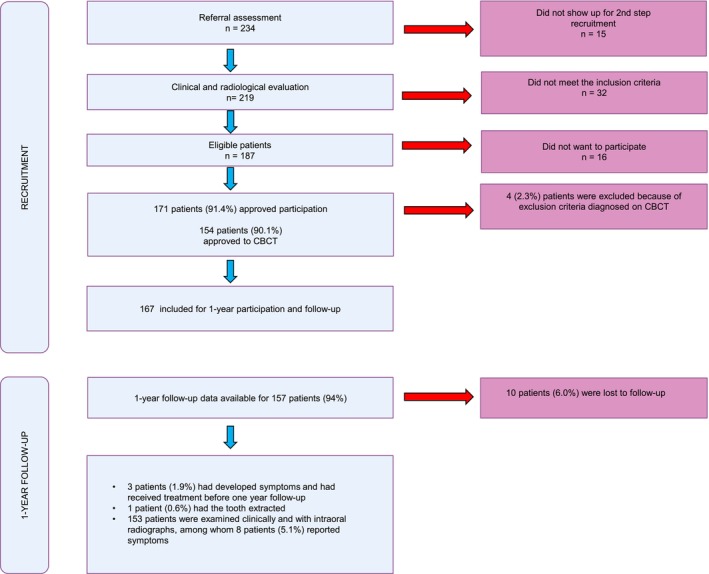
Flow‐chart of study.

A flow chart of the study is exhibited in Figure 1.

### 4.1 Included Patients and Loss to Follow‐Up

Between 2015 and 2023, 234 patients were initially identified as potentially eligible for the study. Of these, 15 patients (6.4%) did not attend the clinical and radiographic examination, and 32 patients (13.7%) were excluded for not meeting the inclusion criteria. A total of 187 patients were eligible, and 171 (91.4%) agreed to participate in the study. Among these, 95 (55.6%) were female. The youngest patient was 25 years and the oldest 83 years of age, while the mean age was 55.7 years. Patient and tooth characteristics are presented in Table 1.

**TABLE 1:** Baseline characteristics of included patients and their teeth.

Variable	All included patients (*n* = 171)	Patients with follow‐up (*n* = 157)
Age	55.7 (13.8) 56 (25;83) (45; 67)	55.1 (13.6) 56 (25;83) (45;66)
Gender		
Female	95 (55.6%)	87 (55.4%)
Male	76 (44.4%)	70 (44.6%)
Tooth group		
Incisor/canine	29 (17.0%)	26 (16.6%)
Molar	103 (60.2%)	96 (61.1%)
Premolar	39 (22.8%)	35 (22.3%)
Jaw		
Mandible	93 (54.4%)	86 (54.8%)
Maxilla	78 (45.6%)	71 (45.2%)
Coronal restoration		
Bridge	2 (1.2%)	1 (0.6%)
Filling	62 (36.3%)	57 (36.3%)
Crown	107 (62.6%)	99 (63.1%)
Post		
No	101 (59.1%)	96 (61.1%)
Yes	70 (40.9%)	61 (38.9%)
Type of post		
Composite post	20 (28.6%)	16 (26.2%)
Metallic post	50 (71.4%)	45 (73.8%)
Number of surfaces of filling		
One	10 (15.4%)	9 (15.3%)
Two	16 (24.6%)	16 (27.1%)
Three	12 (18.5%)	12 (20.3%)
Four	15 (23.1%)	13 (22.0%)
Five	12 (18.5%)	9 (15.3%)

*Note:* For categorical variables *n* (%) is presented. For continuous variables Mean (SD)/Median (Min; Max) (Q1; Q3) is presented.

After a supplementary CBCT (*n* = 154), 4 (2.6%) patients were excluded because the periapical bone destruction breached cortical constrictions and reached the maxillary sinus or mandibular nerve canal. Consequently 167 patients were included for monitoring without intervention and scheduled for a 1‐year follow‐up.

During the review period, three patients (1.8%) withdrew from the study. For unknown reasons, four patients (2.4%) did not respond to our call for follow‐up examination. Another three patients (1.8%) were not recalled due to administrative errors. Consequently, the loss to follow‐up summed up to 10 patients (6.0%).

### 4.2 Clinical Follow‐Up

Among the remaining 157 patients, 3 (1.9%) teeth had developed symptomatic AP and had been treated with either non‐surgical retreatment (1 tooth), surgical retreatment (1 tooth) or extraction (1 tooth) before the recall. Another 1 tooth was extracted due to tooth fracture. The time that had elapsed from baseline to event ranged from 133 to 264 days (Median 206).

Hence, 153 teeth had a 1‐year clinical and radiological follow‐up. Due to, among other factors, the Covid pandemic, the actual follow‐up time deviated from the planned 365 days for several patients. The mean number of days between baseline and follow‐up was 402 (SD 62, Median 391 (Min 301–Max 908)). Among those attending, eight patients (5.2%) recalled some subjective pain or discomfort during the period. They had, however, neither sought nor received any treatment for their ailments.

Consequently, among 157 asymptomatic root‐filled teeth with AP the incidence of AP related to our primary outcome measure of symptomatic conditions was 11 (7.0%). One further tooth was diagnosed with a fracture during clinical examination, making a total of two teeth (1.3%) with our secondary outcome of major clinical events not related to AP during the follow‐up since baseline.

The clinical variables at baseline and follow‐up examinations including frequency of changes are presented in Table 2. The changes were few or small and were not statistically significant (*p* > 0.05). Only four patients (2.6%) had developed clinical signs of apical periodontitis, reporting pain on percussion and/or palpation. However, bleeding on probing (BOP) was found in 35 teeth (23%) in which there had been none registered at baseline, but on the other hand 24 (15.8%) teeth had the reverse: BOP at baseline but not at recall.

**TABLE 2:** Baseline, follow‐up and changes from baseline to follow‐up in clinical findings.

Variable	Baseline (*n* = 171)	Baseline for patients with follow‐up (*n* = 153)	1‐year follow‐up (*n* = 153)	Change from baseline to follow‐up	*p*
Percussion pain/tenderness				Decrease 0 (0.0%)	
No	171 (100.0%)	153 (100%)	151 (98.7%)	Equal 151 (98.7%)	
Yes	0 (0.0%)	0 (0%)	2 (1.3%)	Increase 2 (1.3%)	0.50
Crown pain/tenderness				Decrease 0 (0.0%)	
No	171 (100.0%)	153 (100%)	149 (97.4%)	Equal 149 (97.4%)	
Yes	0 (0.0%)	0 (0.0%)	4 (2.6%)	Increase 4 (2.6%)	0.13
Apical pain/tenderness			*	Decrease 0 (0.0%)	
No	171 (100.0%)	153 (100%)	148 (97.4%)	Equal 148 (97.4%)	
Yes	0 (0.0%)	0 (0%)	4 (2.6%)	Increase 4 (2.6%)	0.13
Pocket depth (mm)			*		
0	2 (1.2%)	2 (1.3%)	1 (0.7%)		
1	45 (26.3%)	40 (26.1%)	37 (24.3%)		
2	93 (54.4%)	82 (53.6%)	93 (61.2%)		
3	24 (14%)	23 (15.0%)	19 (12.5%)		
4	6 (3.5%)	5 (3.3%)	1 (0.7%)	Decrease 39 (25.7%)	
5	1 (0.6%)	1 (0.7%)	0 (0.0%)	Equal 72 (47.4%)	
6	0 (0.0%)	0 (0.0%)	1 (0.7%)	Increase 41 (27.0%)	0.72
Pocket depth (mm)	1.94 (0.80)	1.95 (0.81)	1.91 (0.72)	−0.03	0.72
	2 (0; 5)	2 (0; 5)	(2; 6)	(−0.18; 0.11)	
Bleeding on probing			*	Decrease 24 (15.8%)	
No	124 (72.5%)	112 (73.2%)	101 (66.4%)	Equal 93 (61.2%)	
Yes	47 (27.5%)	41 (26.8%)	51 (33.6%)	Increase 35 (23.0%)	0.15

*Note:* For categorical variables *n* (%) is presented. For continuous variables Mean (SD)/Median (Min; Max)/*n* = is presented. For change in continuous variables Mean (95% CI for Mean using the inversion of Fisher's non‐parametric permutation test)/*n* = is presented. For comparison over time, the Wilcoxon Signed Rank test was used for continuous variables and Sign test was used for categorical variables.

* Due to missing recording variable at follow‐up sum up only to *n* = 152.

### 4.3 Radiographic Observer Variation Analysis

The intra‐ and inter‐observer variation in measuring the size of the bone destructions was small in absolute terms and did not reach statistical significance (Table 3). The variation in recording the other radiological variables is displayed in Table 4. Both observers showed occasional intra‐observer variations in the interpretation of the radiographs. However, after correcting for chance (Gwet's AC1), the agreement varied between 0.75 and 1.0. The analyses of inter‐observer discrepancies also revealed good agreement when corrected for chance for most variables. However, the judgment of the quality of the seal was somewhat of an exception and reached only 0.60.

**TABLE 3:** Intra‐and interobserver agreement of measurements of periapical bone destruction size (sum of 2 perpendicular diameters in mm) on intra‐oral radiographs.

Intra‐observer agreement (*n* = 25)						
Observer	Measurement 1	Measurement 2	Difference Measurement 2–1	Systematic changes	Intra‐observer SD (IISD)	Intraclass correlation coefficient^b^
	Mean (SD)	Mean (SD)	Mean (SD)	*p* ^a^		
	Median (Min; Max)	Median (Min; Max)	Median (Min; Max)			
MSO	7.33 (1.92)	7.38 (1.99)	0.04 (1.22)	0.65	0.85	0.81
	7.20 (3.20; 10.70)	7.60 (3.70; 10.80)	0.20 (−2.90; 2.00)			
JO	6.91 (2.18)	6.84 (2.26)	−0.07 (0.91)	0.73	0.63	0.92
	6.20 (4.20; 11.80)	6.30 [3.40; 11.80]	0.10 (−2.00; 2.50)			

Inter‐observer agreement (*n* = 25)						
Measurement	Observer MSO	Observer JO	Difference JO‐ MSO	Systematic changes	Intra‐observer SD (IISD)	Intraclass correlation coefficient^b^
	Mean (SD)	Mean (SD)	Mean (95% CI Limits of agreement) (SD)			
	Median (Min; Max)	Median (Min; Max)	Median (Min; Max)	*p* ^a^		
	7.33 (1.92) 7.20 (3.20; 10.70)	6.91 (2.18) 6.20 (4.20; 11.80)	−0.42 (−3.53; 2.69) (1.59) −0.30 (−3.90; 2.30)	0.24	—	0.70

^a^ Wilcoxon Signed Rank test is used to test the difference.

^b^ Single measurement, random effect.

**TABLE 4:** Intra‐and interobserver agreement on observations of various categorical variables in intraoral radiographs.

Intra‐observer agreement 1st vs. 2nd observation								
Feature	Observer	1\1*	0\0*	1\0*	0\1*	Agreement (95% CI)	Simple Kappa (95% CI)	Gwet's AC1 (95% CI)
Root resorption	MSO	1	23	1	0	0.96 (0.80, 1.00)	0.65 (0.02, 1.00)	0.95 (0.86, 1.00)
	JO	1	23	0	1	0.96 (0.80, 1.00)	0.65 (0.02, 1.00)	0.95 (0.86, 1.00)
Unfilled root canal	MSO	1	22	1	1	0.92 (0.74, 0.99)	0.46 (−0.18, 1.00)	0.91 (0.77, 1.00)
	JO	2	23	0	0	1.00 (0.86, 1.00)	1.00 (1.00, 1.00)	1.00 (1.00, 1.00)
Fractured instrument	MSO	1	24	0	0	1.00 (0.86, 1.00)	1.00 (1.00, 1.00)	1.00 (1.00, 1.00)
	JO	1	24	0	0	1.00 (0.86, 1.00)	1.00 (1.00, 1.00)	1.00 (1.00, 1.00)
Overfilling	MSO	4	17	1	3	0.84 (0.64, 0.95)	0.57 (0.19, 0.94)	0.75 (0.50, 1.00)
	JO	3	22	0	0	1.00 (0.86, 1.00)	1.00 (1.00, 1.00)	1.00 (1.00, 1.00)
Underfilling	MSO	1	22	2	0	0.92 (0.74, 0.99)	0.47 (−0.13, 1.00)	0.91 (0.77, 1.00)
	JO	5	19	1	0	0.96 (0.80, 1.00)	0.88 (0.66, 1.00)	0.94 (0.82, 1.00)
Defective seal	MSO	5	18	2	0	0.92 (0.74, 0.99)	0.78 (0.50, 1.00)	0.87 (0.70, 1.00)
	JO	6	18	1	0	0.96 (0.80, 1.00)	0.90 (0.70, 1.00)	0.93 (0.81, 1.00)

Inter‐observer agreement MSO vs. JO							
Feature	1/1**	0/0**	1/0**	0/1**	Agreement (95% CI)	Simple Kappa (95% CI)	Gwet's AC1 (95% CI)
Root resorption	1	23	1	0	0.96 (0.80, 1.00)	0.65 (0.02, 1.00)	0.95 (0.86, 1.00)
Unfilled root canal	1	22	1	1	0.92 (0.74, 0.99)	0.46 (−0.18, 1.00)	0.91 (0.77, 1.00)
Fractured instrument	1	24	0	0	1.00 (0.86, 1.00)	1.00 (1.00, 1.00)	1.00 (1.00, 1.00)
Overfilling	3	20	2	0	0.92 (0.74, 0.99)	0.71 (0.33, 1.00)	0.89 (0.74, 1.00)
Underfilling	3	19	0	3	0.88 (0.69, 0.97)	0.60 (0.22, 0.99)	0.83 (0.63, 1.00)
Defective fill	4	15	3	3	0.76 (0.55, 0.91)	0.40 (0.01, 0.80)	0.60 (0.28, 0.92)

*Note:* 1/1*, recorded at both observations; 1/1**, recorded by both observers. 0/0*, not recorded at any observation; 0/0**, not recorded by any observer. 1/0*, recorded at first observation but not at second observation; 1/0**, recorded by MSO but not by JO. 0/1*, not recorded at first observation but at second observation; 0/1**, not recorded by MSO but by JO.

### 4.4 Radiographic Follow‐Up

Change in the size of periapical bone destruction (third outcome) was small (M = 0.059 mm) and the mean size of the bone destruction increased from 7.25 mm (SD 2.03) to 7.31 mm (SD 2.33) which was not statistically significant (Table 5). Only in 3 teeth (2.0%) did the size of bone destruction increase ≥ 4 mm, while 2 teeth had a decreased size ≥ 4 mm (1.3%). The change in size of bone destruction is exhibited in Table 5.

**TABLE 5:** Changes from baseline to follow‐up in radiographic observations.

				Change from Baseline to 1‐year follow‐up	
Variable	Baseline (*n* = 171)	Baseline for patients with follow‐up (*n* = 153)	1‐year follow‐up (*n* = 153)		*p*
Root resorption				Decrease 3 (2.0%)	0.73
No	164 (95.9%)	150 (98.0%)	148 (96.7%)	Equal 145 (94.8%)	
Yes	7 (4.1%)	3 (2.0%)	5 (3.3%)	Increase 5 (3.3%)	
Unfilled root canal				Decrease 4 (2.6%)	0.12
No	161 (94.2%)	145 (94.8%)	138 (90.2%)	Equal 138 (90.2%)	
Yes	10 (5.8%)	8 (5.2%)	15 (9.8%)	Increase 11 (7.2%)	
Fractured instrument				Decrease 3 (2.0%)	0.63
No	163 (95.3%)	146 (95.4%)	148 (96.7%)	Equal 149 (97.4%)	
Yes	8 (4.7%)	7 (4.6%)	5 (3.3%)	Increase 1 (0.7%)	
Overfilling				Decrease 4 (2.6%)	1.00
No	151 (88.3%)	135 (88.2%)	136 (88.9%)	Equal 146 (95.4%)	
Yes	20 (11.7%)	18 (11.8%)	17 (11.1%)	Increase 3 (2.0%)	
Underfilling				Decrease 16 (10.5%)	0.35
No	143 (83.6%)	129 (84.3%)	134 (87.6%)	Equal 126 (82.4%)	
Yes	28 (16.4%)	24 (15.7%)	19 (12.4%)	Increase 11 (7.2%)	
Defective fill				Decrease 7 (4.6%)	0.0002
No	118 (69.0%)	105 (68.6%)	85 (55.6%)	Equal 119 (77.8%)	
Yes	53 (31.0%)	48 (31.4%)	68 (44.4%)	Increase 27 (17.6%)	
Size of bone destruction (mm)	7.35 (2.11)	7.25 (2.03)	7.31 (2.33)	0.059 (−0.243:0.357)	0.99*
	7.4 (1.5:14.4)	7.3 (1.5;14.4)	7.2 (1.5:15.6)		

*Note:* For categorical variables *n* (%) is presented. For continuous variables Mean (SD)/Median (Min:Max)/*n* = is presented. For change in continuous variables Mean (95% CI for Mean using the inversion of Fisher's non‐parametric permutation test)/*n* = is presented. For comparison over time, the Willcoxon signed Rank test was used for continuous variables and Sign test was used for categorical variables.

* The effect size was 0.03 estimated by using the Cohen method (difference in mean/SD at first timepoint).

Also, the changes of the other radiological variables over the follow‐up period are displayed in Table 5. Changes were generally few and did not reach statistical significance except for the estimation of density of seal which was more often deemed as poor at follow‐up compared to baseline (*p* < 0.001).

## 5 Discussion

The incidence of clinically negative events (pain and interventions) was low (13 teeth, 8.3%) during the follow‐up period in this prospective longitudinal study of asymptomatic root‐filled teeth with AP.

Previous studies on this topic are rare. The risk of an exacerbation (also named flare‐up), where an asymptomatic lesion suddenly becomes painful, has been estimated to be 5.8% over a period of 20 years in a population treated at a university clinic (Yu, Messer, Yee, and Shen 2012). A low risk of painful exacerbations (1%–2%) was also previously reported from a cohort of 1032 root‐filled teeth followed over time by Van Nieuwenhuysen et al. (1994). Very little is known about potential risk factors for flare‐ups (exacerbation) of asymptomatic lesions. In the report from Singapore where 127 patients with 185 non‐healed root‐filled teeth were recruited (Yu, Messer, Yee, and Shen 2012), the incidence of discomforting clinical events was significantly associated with female patients, treatments involving a mandibular molar or maxillary premolar, and preoperative pain.

The fact that in our study, more patients had experienced symptoms within 1 year than in other studies for a significantly longer time, suggests that our collected cohort represents a different sample. In both studies Yu, Messer, Yee, and Shen 2012 and Van Nieuwenhuysen et al. (1994), the patients' root canal treatments had been carried out at specialist and university clinics, while our patients were collected from a group of patients where the root canal treatments had been completed in general dentistry and were referred because the dentist assessed the result as non‐healing.

There is thus an initial bias in recruitment to the study since eligible patients were not a randomized sample of the patients with root‐filled teeth with persistent AP.

A strength of this study is the use of a clinically well‐defined patient cohort, with combined clinical and radiographic assessment, which enhances internal validity. However, the non‐randomised recruitment process introduces a risk of selection bias, as all patients had received their primary root canal treatment in general dental practice and were subsequently referred by general practitioners based on an assessment of non‐healing. Patients treated by endodontic specialists were not included, which may limit the generalizability of the findings to other clinical settings. In addition, both symptom reporting and radiographic interpretation involve a degree of subjectivity and observation bias. Despite these limitations, the study provides valuable data on the natural course of persistent apical periodontitis in cases where no further treatment is undertaken—an area that remains underreported in the literature.

The low number of events in our present study makes a valid statistical analysis of potential risk factors not possible. But, considering that, despite the low incidence of events, events already exceed what was previously reported after a significantly longer follow‐up period, there are good prospects for identifying risk factors if the study is extended over time.

During the follow‐up, changes in the estimated size of the apical bone destructions as interpreted in intra‐oral radiographs were small. Most changes were within what could be expected just from observer variation (a decrease or increase < 4 mm) (Table 3). Only three teeth exhibited greater expansion while two teeth actually became > 4 mm smaller. The number of teeth with significant changes in size of the destruction was too few to allow any inference with potential prognostic factors.

Tsesis et al. (2013) retrospectively evaluated the radiologic appearance changes using the periapical index (PAI) score (Ørstavik et al. 1986) between two consecutive periapical surveys of at least a 4‐year interval in 74 patients with 200 root‐filled teeth with AP. They found that 57 (28.5%) lesions remained unchanged, 103 (51.5%) lesions worsened (PAI score increased), and 40 (20%) lesions improved (PAI score decreased). Poor root canal filling and poor restoration were found to significantly adversely affect the radiographic appearance of AP.

In a study from a university clinic in Singapore Yu et al. retrospectively evaluated (4–21 years) after first recall, 151 AP lesions in root‐filled teeth (Yu, Messer, Shen, et al. 2012). Eighty‐six lesions (57.0%) improved, 18 (11.9%) remained unchanged, and 47 (31.1%) deteriorated since treatment. Potential predictors for lesions that did not improve included lesion size, pain on biting at recall examination, history of a post‐root‐filling flare‐up, and a non‐ideal root‐filling length. Lesions that had persisted for a longer period also appeared less likely to be improving. Based on these parameters the researchers presented a risk score algorithm (Deterioration Risk Score) ranging from very low risk to very high risk for the lesion to increase in size over time (Yu et al. 2014). The authors pointed out the need for further research on this topic in order to validate the algorithm.

The changes in size of the periapical bone over time may need a more sensitive tool for analyses than intra‐oral radiographs (Boubaris et al. 2021). A majority of the patients enrolled in our study accepted an invitation to a CBCT examination at baseline and we are planning a 5‐year follow‐up using CBCT which will permit a more precise estimation of any changes in lesion size.

The changes of other variables assessed on intraoral radiographs, except for quality of seal, did not elicit any statistically significant change. Most of the observed differences may, to a big extent, be explained by observer variation. However, the significant increase in the number of teeth assessed to exhibit a defective seal, is probably not due to random variation but rather a systematic shift over time in the observers' criteria for what is defective or not. The difficulty in maintaining a strict criterion on root filling quality has been shown before (Reit and Hollender 1983).

There is a concern about the possible negative impact on systemic health by endodontic inflammatory disease in general and apical periodontitis especially. It therefore may appear unethical to suggest to patients not to treat a tooth diagnosed with AP. It is then important to bear in mind that this condition is very common all over the world (Pak et al. 2012; Jakovljevic et al. 2020; Tibúrcio‐Machado et al. 2021) and much research has shown that many dentists neither suggest nor perform treatment as long as the patient is symptom‐free (Petersson et al. 1991; Kirkevang et al. 2014; Olsson et al. 2024). Another aspect is that even if there was a consensus that treatment should be instituted, it would require substantial financial and personal resources. Indeed, to such an extent that pursuing it would be virtually impossible.

However, the general health of the patients in our study needs to be continuously checked by updating their medical history. Since the mean age of patients at inclusion was almost 55 years, it is reasonable to expect some of them to develop severe disease during the course of the study. It was noted, at the clinical and radiological controls, that two patients now were suffering from disease (one heart attack and one of pancreatic cancer) that would render a classification III according to the ASA.

Uncertainties are present in all clinical settings (Han et al. 2011). The outcome of root canal treatment has been extensively researched and we know reasonably well about the probability of the outcomes and the influence of various factors (Gulabivala and Ng 2023). However, in many cases the conditions are special, unusual or difficult to assess and therefore it is still difficult to comment with certainty on the prognosis in an individual case. By pursuing more evidence, we try to reduce this type of fundamental uncertainty. The role of microorganisms in the root canal system and persistent AP is well established. Therefore, when leaving the condition untreated it is expected that some patients may develop symptoms or that lesions will increase in size. By pursuing research like ours, we try to reduce fundamental uncertainty by establishing the probabilities of various negative events (Kvist and Hofmann 2023).

In addition to being able to establish probability figures for different outcomes of leaving a condition untreated, it would be of great value to also be able to identify anamnestic, clinical or radiological prognostic factors. We have recorded a variety of such different potential variables (gender, age, tooth group, root canal quality, type of restoration, etc.) But in order to be able to make statistical calculations, a sufficiently large number of events is also required.

At this 1‐year follow‐up, only 11 teeth developed symptoms that could be related to AP. This is not enough to form the basis for deeper statistical analysis. With a longer follow‐up period, it is likely that more patients will develop symptoms, which makes such an analysis possible.

A significant disadvantage of this cohort study is that there is no control group. Such a candidate could be recruited in at least two ways. A first possibility would be to recruit a parallel group, preferably matched in terms of age, sex, tooth group restoration, root filling quality but where there are no signs of AP. Even teeth without AP are lost at a steady rate (Dawson et al. 2024) and there are also reports suggesting that pain from root canal filling cannot always be linked to the presence of AP (Jonsson Sjögren et al. 2019). Also, AP may develop over time and result in apical bone destruction.

But better still would be a randomised clinical trial in which root‐filled teeth with asymptomatic AP were allocated to either endodontic retreatment or monitoring. Outcome measures of interest would be general health, dental survival, symptom freedom, costs and patient satisfaction. Results from this prospective cohort study without a control group can thereby form part of the basis for planning and, for example, calculation of statistical power and necessary sample size.

## 6 Conclusion

Our study corroborates previous studies and the general opinion that symptom‐free root‐filled teeth with apical periodontitis in the short term rarely lead to an exacerbation that makes treatment necessary or that the bone destruction increases greatly in size. But to identify potential risk factors, a longer follow‐up period is required.

## Author Contributions

All authors contributed to the study conception and design and read and approved the final manuscript. The researchers within the Endodontic Research Collaboration in Scandinavia contributed to this study. Collaborators: L. Bjørndal, V.S. Dawson, H. Fransson, F. Frisk, M. Markvart, F. Mota de Almeida, M. Pigg, D. Sebring and E. Wigsten.

## Consent

Written informed consent was obtained from all individuals included in the study.

## Conflicts of Interest

The authors declare no conflicts of interest.

## Data Availability

Data available on request from the authors.
